# Illness Perceptions, Self-Care Management, and Clinical Outcomes According to Age-Group in Korean Hemodialysis Patients

**DOI:** 10.3390/ijerph16224459

**Published:** 2019-11-13

**Authors:** Sisook Kim, Eunhye Kim, Eunjung Ryu

**Affiliations:** 1 Department of Nursing, Kyungmin University, Uijeongbu-si 11618, Korea; kmkss@kyungmin.ac.kr; 2 Department of Head & Neck—Thyroid Center, Samsung Medical Center, Seoul 06351, Korea; eh0502.kim@samsung.com; 3 Department of Nursing, Chung-Ang University, Seoul 06974, Korea

**Keywords:** age difference, hemodialysis, illness perception, self-care management, clinical outcomes

## Abstract

Illness perception, formed in social-cultural contexts, is the driving force for health behavior. Age difference can affect health outcomes due to its association with socioeconomic status. The purpose of this study is to determine the relationship between illness perception, self-care management, and clinical outcomes according to the age group in hemodialysis patients. A cross-sectional study was conducted. Using the Brief Illness Perception Questionnaire (BIPQ) and Patient Activation Measure (PAM) 13, clinical outcomes, such as serum phosphorus, potassium, hemoglobin, and albumin were investigated in maintenance hemodialysis patients. Illness perception, self-care management, and clinical outcomes in 189 hemodialysis patients were different according to age group. Younger hemodialysis patients had the lowest illness perception and the highest serum phosphorus. Illness perception was associated with self-care management and clinical outcomes. After the adjusted age, the association between illness perception and self-care management and the association between illness perception and phosphorus were reduced, while the association between illness perception and potassium increased. Uncontrolled phosphorus in younger hemodialysis patients can increase the risk of cardiovascular complications and mortality. To improve self-care management and clinical outcomes in hemodialysis patients, reconstruction, or expansion of illness perception needs to be differentiated according to age group.

## 1. Introduction

Since the 1980s, the role of self-care management has been emphasized by the rapid increase in the number of outpatient hemodialysis patients [1]. Self-care management, including regular dialysis, fluid and diet restriction, medications, and vascular monitoring, is essential for health in daily life. However, many hemodialysis patients are at risk of complications or high mortality because they deliberately omit or shorten dialysis treatment time [2] and fail to maintain proper management of water and diet [3]. The quality of health and survival depends on how hemodialysis patients manage their chronic illness, dialysis, and everyday lifestyle throughout their lives. However, in Korea for example, the 5-year survival rate is only 48%, even though 80% of hemodialysis patients have independent self-care [4]. 

Self-care management and hemodialysis skills are related to demographic variables such as age, sex, marriage, and employment [5]. Considering the impact of self-care management on health outcomes, differential self-care management could reduce the gap—including that related to demographic variables—in health outcomes. Therefore, there is a need for a self-care management strategy that takes into account the demographic variables of hemodialysis patients. 

Illness perception is a subjective view that focuses on one’s experience and condition due to an illness, and it offers insight into how health behaviors of chronic patients can be sustained [6]. It is influenced by cognitive and emotional aspects, such as the expected timeline of the illness, life consequences due to the illness, how the illness is controlled or treated, the identity and cause of the illness, and emotions, such as fear or anxiety related to the illness [7]. Individual illness perception acts as a driving force and starting point for coping with and action plans for illness [8], and it is associated with psychosocial and clinical outcomes in hemodialysis patients [9]. Hemodialysis patients with high control of illness perception and low perceived consequences for their lives were found to have low depression [10]. For hemodialysis patients with high illness perception of chronic timelines, inter-dialytic weight control was good [11].

Illness perception based on the social-cultural context demonstrates age-related differences; younger patients have the confidence to successfully cope with the illness while not giving up their fears related to the illness, whereas older people adhered only when they felt they needed direct self-care, and were less angered because of the illness [12]. Hemodialysis patients had a high perception of the consequence that their illness and treatment interfered with daily life after the start of dialysis, but with increasing age, the perception of consequence and emotional reactions decreased [13]. Although illness perception in hemodialysis patients is related to self-care management and age, in many studies, the age of hemodialysis patients is only described as a characteristic of the study sample or treated as a fixed variable (Table 1). 

Self-care management based on age could be a multi-faceted strategy suitable for the rapid change in the healthcare environment. The assessment of illness perception according to age is expected to reduce the gap between self-care management and health outcomes. Therefore, this study aimed to determine the relationship of illness perception, self-care management, and clinical outcomes according to age group among hemodialysis patients.

## 2. Materials and Methods

### 2.1. Design and Ethics

**Figure 1:**
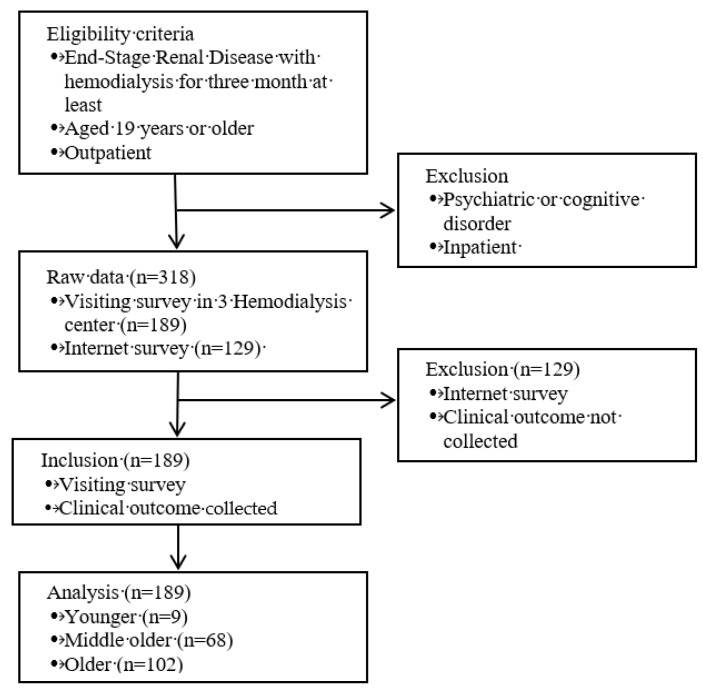
Flow of this study.

This is a secondary data analysis of a cross-sectional study that involved outpatients from three hemodialysis centers in Korea. Raw data were collected between January 1 and March 31, 2017 [14]. Figure 1 shows the flow of this study. This study was approved by the author’s institutional ethics committee—IRB, approval number: 1041078-201907-HRBM-222-01. While collecting raw data, the study was performed in accordance with the provisions of the Helsinki Declaration and subsequent amendments.

### 2.2. Participants and Sample Size

Eligibility criteria included the following: subjects diagnosed with ESRD (End-Stage Renal Disease) and treated with hemodialysis for at least three months, aged 19 years or older, lived at home, were able to read and write, had no psychiatric or cognitive disorders, and were willing to participate in the study. By referring to sociodemographic criteria and precedent studies, the participants were classified into three age groups: the younger group (<50 years), middle-older group (from 50 to under 65 years), and older group (≥65 years) [15,16].

The sample in this study comprised 189 patients. This was more than the minimum required sample size of 159 for the two-tailed ANOVA of the three groups, with an alpha of 0.05, power of 0.8, and medium effect size of 0.25 using G*power 3.1.9.4.18 [17].

### 2.3. Measures

Socio-demographic and disease-related characteristics. Data on age, sex, education level, monthly income, monthly medical expenditure, diabetes mellitus status, hypertension status, kidney transplantation history, and duration of hemodialysis were collected from the maintenance of hemodialysis patients. 

Illness perception was assessed using the Brief Illness Perception Questionnaire (BIPQ) [18]. This tool consists of eight items related to illness perception (identity, timeline, consequence, treatment control, personal control, emotion and concern, and understanding of illness) and one open item (cause of illness). In this study, only the eight items were used, excluding the open item. Each item was scored on a 0–10 Likert scale, with higher scores indicating higher illness perception. The Cronbach’s α of BIPQ was 0.766 in this study. 

Self-care management was assessed using the Patient Activation Measure (PAM) 13 [19]. This tool consists of 13 items in four categories: (1) believing the patient role is important, (2) having the knowledge and confidence necessary to take action, (3) actually taking action to maintain and improve one’s health, and (4) continuing the course even under stress. Each item was scored on a 1–4 Likert scale, with higher scores indicating higher knowledge, skills, and confidence about self-care management. The PAM 13 scores can be converted to scores ranging between 0 (lowest activation) and 100 (highest activation) for comparison. The mean score for the general adult population at the time of development was 61.9, whereas the mean score for hemodialysis patients in this study was 59.5. The Cronbach’s α of PAM 13 was 0.865 in this study. 

Based on precedent studies and international guidelines related to self-care management in hemodialysis patients [20,21,22], data on serum phosphorus, potassium, hemoglobin, and albumin levels were collected as clinical outcomes. Medical records were reviewed and the latest results from within the past month before dialysis were collected.

### 2.4. Data Analysis

The collected data were analyzed using SPSS 23.0. Because of the small number of participants in the younger group (*n* = 19), differences in sociodemographic and disease-related characteristics were analyzed using Fisher’s exact test and have been described in terms of frequencies and percentages. Illness perception, self-care management, and clinical outcomes according to the age group were analyzed using the Kruskal–Wallis test and the Bonferroni correction method for post-hoc analysis, and are described in terms of means and standard deviations. The correlations among illness perception, self-care management, and clinical outcomes according to the age group were analyzed using the Pearson’s correlation coefficient and partial correlation coefficient. 

## 3. Results

### 3.1. Descriptive Analysis and Difference in Study Variables

In total, there were 189 participants. The median ages were 45 years for the young group, 58 for the middle group, and 73 for the older group. There were no significant differences in gender, education level, and average monthly income among the three groups. In all three groups, there were slightly more men than women, participants were mostly high school graduates, and their socio-economic level was low, with monthly income below 1,000,000 KRW. Monthly medical expenses differed according to age group, and the older the participants, the higher their medical expenses.

The duration of hemodialysis was mostly less than 10 years, and less than 5 years was the most common in all three groups. There was no difference in the percentage of hypertension and diabetes in the three groups. Hypertension and diabetes were high in the younger group. There was little experience of kidney transplantation (Table 2).

### 3.2. Differences in Study Variables According to Age Group

Participants were highly perceptive of the timeline and treatment control for the illness, with the lowest perception being of personal control. There were significant differences in illness perception, self-care management, and clinical outcomes according to age group (Table 3).

While the illness perception of the middle older group was highest (H = 8.764, *p* = 0.013), the younger group had a lower perception of “personal control” of illness than did the older and middle older groups (H = 11.852, *p* = 0.003). Self-care management was higher in the older group, but there was no significant difference among the three groups. In self-care management, “believing that the patient’s role is important” (H = 8.007, *p* = 0.018) and “maintaining behavioral change” (H = 9.124, *p* = 0.010) was significantly higher among the older group than the younger.

Serum phosphorus and potassium were significantly different according to age group. The serum phosphorus (H = 15.957, *p* = 0.000) and potassium (H = 7.075, *p* = 0.029) of the younger group were outside the normal range and higher than in the older and middle older groups. Hemoglobin and albumin did not differ by age group and were within the normal range of hemodialysis patients.

### 3.3. Correlations among Illness Perception, Self-Care Management, and Clinical Outcomes according to Age Groups 

Table 4 shows that as the age of the hemodialysis group increased, self-care management improved (r = 0.148, *p* = 0.043) and serum phosphorus (r = −0.284, *p* = 0.000) and potassium (r = −0.223, *p* = 0.002) decreased. As illness perception increased, self-care management improved (r = 0.282, *p* = 0.000) and potassium (r = 0.208, *p* = 0.004) and hemoglobin (r = −0.243, *p* < 0.01) increased. In contrast, as illness perception increased, phosphorus decreased (r = −0.257, *p* = 0.000).

After adjusting for age group, the positive association between illness perception and self-care management (r = 0.268, *p* = 0.000), and the negative association of illness perception and phosphorus (r = −0.233, *p* = 0.001) decreased. On the other hand, the positive association between illness perception and potassium increased (r = 0.244, *p* = 0.001), and the significant association between illness perception and hemoglobin disappeared (Table 4). The change after the adjusted age group may be explained some effect of age group to the association of illness perception, self-care management, and clinical outcomes.

## 4. Discussion

This study aimed to identify the differences in illness perception, self-care management, and clinical outcomes according to the age group of hemodialysis patients. Chronological age is a major risk factor for chronic disease and mortality, and it is natural for older people to have higher mortality rates than younger people, even without disease. However, age differences in hemodialysis patients may affect health outcomes as they relate to education levels, income, daily living functions, self-efficacy, and dietary control [30]. The demographic and disease-related characteristics of the participants in this study (gender, education, income, duration of dialysis, hypertension, and diabetes) did not vary among the three groups (younger, middle-older, and older). However, there were significant differences in illness perception, self-care management, and clinical outcomes among the three age groups.

First, the participants’ illness perception was highest in order of timeline, treatment control, and concern, which is consistent with previous findings related to illness perception in hemodialysis patients [11,33]. When confronted with the illness, an individual respond to the illness cognitively and emotionally giving personal meaning to the illness; this is their illness perception [8,34]. Chronic disease patients often have a higher perception of their illness’s expected course over their timeline, but myocardial infarction or neurology patients or inpatients may have higher illness perception of treatment control, understanding, consequences, or concerns than of their timelines [35]. Hemodialysis patients are aware that their lives are significantly influenced by dialysis, but they cannot live without it, so they are forced to live with the physical limitations caused by their treatment, and may feel helpless [36]. They routinely experience fears related to death and an uncertain future and struggle with feelings such as anger, guilt, and depression regarding their illness [37]. Illness perception—which predicts chronic disease duration, affects patient belief that their illness will be controlled by treatment, and concerns life consequences—is thought to reflect the conditions and experiences of hemodialysis patients. 

The younger group’s illness perception was lower than of the older group, and serum phosphorus in younger patients was higher than the normal range in hemodialysis. Elevated serum phosphorus in hemodialysis patients implies an increased risk of cardiovascular disease and mortality due to calcium-phosphorus complexities as well as skeletal calcification [22]. At the same time, serum phosphorus is a major indicator of self-care management that can be controlled by diet and medication [24]. Younger patients have an advantage when it comes to obtaining knowledge of phosphorus control compared to older patients. However, younger patients’ phosphorus increases are caused in part by eating snacks, dining out, and an over-absorption of protein [31].

What causes inconsistencies in knowledge and behavior of phosphorus control in adolescent hemodialysis patients? Younger patients had much higher psychosocial stressors than physiology stressors, but according to the stages of early adult development, they may focus more on their appearance and physical strength than be concerned with treatment [38]. Another possible cause is that the depression of young hemodialysis patients is associated with high emergency room visits, hospitalizations, and mortality rates, despite their lower medical comorbidity than older patients [39]. In addition, age-related illness perception can lead to differences in health behaviors. The relationship between illness perception, self-care management, and phosphorus was weakened when the age group was adjusted in this study. This reduced relationship between illness perception, self-care management, and phosphorus is explained by the adjusted age. Older hemodialysis patients demonstrated no differences in their perception of the illness’s threat to their health compared to the healthy older group, while younger patients perceive the illness as a loss and challenge and either emotionally cope with it or avoid it [40]. In hemodialysis patients, adherence to treatment is a result of the perception that treatment instructions are not overly difficult, and non-adherence occurs when one perceives the consequence of illness directly and negatively [41]. Therefore, for younger hemodialysis patients, it may be difficult to maintain healthy behaviors if they are not fully aware of treatment recommendations [42].

Illness perception of “personal control” was low among all age groups and the lowest among younger adults. Perception of control in a dependent and limited hemodialysis life would be difficult. However, “personal control” could benefit the self-care management of hemodialysis patients by enhancing their knowledge, experience, and adaptation [13]. Fortunately for the patients, illness perception is not fixed or immutable. The perception of personal control increased and illness perception of consequences and emotions decreased as treatment progressed and patients adapted [33]. Thus, reconstruction or reinforcement of illness perception could serve as a new starting point and driving force for continuing self-care management. 

Decreasing renal function with age is a well-known phenomenon and the average age of hemodialysis patients in global studies was 63.9 years (standard deviation: 15.7) [43]. Increases in lifespan have led to a rise in research on elderly hemodialysis patients. In recent decades, there were 1,110 and 28,707 studies of hemodialysis patients according to a search for the words “younger” and “elderly” on PubMed.com, respectively. However, according to the results of this study, the self-care management and clinical outcomes of these younger patients were worse than that of older patients. And this is indicative of concerns about the future health of young hemodialysis patients. Because younger hemodialysis patients do not make up the majority of the population on hemodialysis, more attention and differential self-management are required. In today’s fast-changing society, the health outcomes of hemodialysis patients are influenced by sociocultural needs and prioritizing health care systems. The attitude of the healthcare staff also plays an important role in hemodialysis patients’ illness perception and health behavior. The health outcome of younger hemodialysis patients is affected by a healthcare environment prone to alienation. Therefore, more research is needed on how to support and care for younger patients. The identification of illness perception by age is expected to contribute to reducing the gap in self-care management and health outcomes.

## 5. Limitation

The main limitation of this study is that there were not enough samples indicating age groups and illness perception of hemodialysis patients in Korean culture. Subsequent research should be conducted with a sufficient number of younger patients and repeat studies through parametric analysis. Hypertension and diabetes are the main comorbidities related to the mortality rate of ESRD patients, but the three groups in this study had a higher rate without hypertension blood pressure, and there was a low level of diabetes in the older group. As the participants in this study were outpatients, more severely ill hemodialysis patients may have been missed. 

## 6. Conclusions

Illness perceptions in hemodialysis patients varied according to age group. Illness perception is not directly related to mortality in this study, but illness perception does relate to self-care management and health outcomes. Thus, assessment and evaluation of illness perception by age may contribute to the health promotion of hemodialysis patients by providing priorities and specificities for self-care planning and management. Low illness perception and self-care management in young adult hemodialysis patients may represent a crisis for health outcomes. Strategies to expand or reconstruct illness perception would contribute to age-specific self-care management and reduce the gaps in health outcomes.

## Figures and Tables

**Table 1:** Relationship between age and variables in hemodialysis patients.

Author (Year of Publication)	Country of Origin	Sample	Age	Association with Age and Variables	Operational Definition	Assessment Based On…
Ahrari et. al. (2014) [23]	Iran	237	46.1 ± 15.4	↑	Frequency and degree of adherence	Diet, fluid,
Atashpeikar et al. (2012) [5]	Iran	115	50.2 ± 15.4	↑	Self-care	Vascular access, diet, general care
Cupisti et. al. (2012) [24]	Italy	119	62 ± 13	↑	Phosphorus control	Serum phosphorus, phosphorus knowledge
Kim and Evangelista (2010) [25]	USA	151	51.9 ± 15.6	↑	Adherence	HD attendance, medication,
Li et. al. (2014) [26]	China	216	53.16 ± 12.86	↓	Self-management	Problem solving
Naalweh et. al. (2017) [27]	Palestine	220	56.82 ± 14.51	↑	Adherence	Medication, fluid restriction, diet recommendation, behavior
Nah et al. (2019) [28]	UK	70	60.1 ± 15.9	↓	Physical activity	Vigorous-moderate-intensity activity; walking and sitting
Natashia et. al. (2019) [29]	Indonesia	145	54.7 ± 12.2	↑	Self-management	Self-care
Sugisawa et. al. (2019) [30]	Japan	6644	66.5	↑	Dietary Restrictions	self-efficacy for dietary restrictions, dietary restrictions
Takayama et. al. (2015) [31]	Japan	331	63.2	↑	Control of calcium and phosphorus	Serum phosphorus and calcium, Prescription of phosphorus binder
Tohme et. al. (2017) [32]	USA	286	64 (IQR: 56–73)	↑	Adherence	Missed dialysis, abbreviated dialysis

**Table 2:** Differences in socio-demographic and disease-related characteristics according to age group in hemodialysis patients (*n* = 189).

Characteristics	Categories	Younger (*n* = 19)	Middle Older (*n* = 68)	Older (*n* = 102)	Fisher’s Exact Test	*p*
Age, Median (range)	Year	45.0 (34–49)	58.0 (50–64)	73.0 (65–91)		
Gender, n (%)	Male	10 (52.6)	42 (61.8)	57 (55.9)	0.835	0.694
	Female	9 (47.4)	26 (38.2)	45 (44.1)		
Education, n (%)	Under elementary	3 (15.8)	7 (10.3)	23 (22.5)	6.963	0.319
	Middle school	3 (15.8)	21 (30.9)	23 (22.5)		
	High School	10 (52.6)	29 (42.6)	36 (35.4)		
	College or higher	3 (15.8)	11 (16.2)	20 (19.6)		
Income, Monthly (1000 KRW), n (%)	<1000	10 (52.6)	29 (42.6)	54 (52.9)	5.023	0.281
	1000–2000	7 (36.9)	18 (26.5)	28 (27.5)		
	>2000	2 (10.5)	21 (30.9)	20 (19.6)		
Medical Cost, Monthly (1000 KRW), n (%)	<100	8 (42.1)	21 (30.9)	15 (14.7)	10.219	0.028
	100–500	10 (52.6)	43 (63.2)	80 (78.4)		
	>500	1 (5.3)	4 (5.9)	7 (6.9)		
Hemodialysis Period (year), n (%)	<5	12 (63.2)	36 (52.9)	66 (64.7)	5.237	0.255
	5–10	7 (36.8)	22 (32.4)	25 (24.5)		
	>10	0 (0)	10 (14.7)	21 (10.8)		
Hypertension, n (%)	Yes	9 (47.4)	22 (32.4)	37 (36.3)	1.500	0.460
	No	10 (52.6)	46 (67.6)	65 (63.7)		
Diabetes Meletus, n (%)	Yes	13 (68.4)	39 (57.4)	46 (45.1)	4.708	0.094
	No	6 (31.6)	29 (42.6)	56 (54.9)		
Kidney Transplantation History, n (%)	Yes	0 (0)	4 (5.9)	0 (0)	5.824	0.027
	No	19 (100.0)	64 (94.1)	102 (100.0)		

KRW, Korean Won.

**Table 3:** Difference of illness perception, self-care management, and clinical outcomes according to age groups in hemodialysis patients (*n* = 189).

Variables	Younger ^a^	Middle Older ^b^	Older ^c^	H	*p*	Post Hoc
Illness Perception	5.40 ± 1.83	6.63 ± 1.58	6.41 ± 1.36	8.764	0.013	a < b
Timeline	6.79 ± 3.19	8.04 ± 2.16	7.56 ± 2.57	2.020	0.364	
Treatment Control	5.89 ± 2.45	7.18 ± 2.27	7.03 ± 2.50	4.027	0.134	
Consequence	5.74 ± 2.75	6.78 ± 2.47	6.70 ± 2.52	2.777	0.249	
Concern	5.37 ± 2.79	6.57 ± 2.68	6.28 ± 2.67	3.064	0.216	
Understanding	5.05 ± 2.01	6.43 ± 2.35	5.87 ± 2.56	5.851	0.054	
Identity	5.00 ± 2.00	6.29 ± 2.54	5.91 ± 2.45	5.173	0.075	
Emotion	4.95 ± 2.01	5.54 ± 2.75	5.71 ± 2.23	2.197	0.333	
Personal Control	4.42 ± 1.74	6.21 ± 2.28	6.25 ± 2.27	11.852	0.003	a < b < c
Self-Care Management (transformed to 100)	55.81 ± 13.53	57.84 ± 11.46	61.33 ± 14.71	4.419	0.110	
Believes Active Role Important	2.97 ± 0.59	3.1 ± 0.58	3.29 ± 0.57	8.007	0.018	
Confidence and Knowledge to Take Action	2.86 ± 0.43	2.92 ± 0.39	2.99 ± 0.55	1.686	0.430	
Taking Action	2.76 ± 0.57	2.85 ± 0.5	2.96 ± 0.59	2.558	0.278	
Staying the Course under Stress	2.53 ± 0.56	2.71 ± 0.57	2.91 ± 0.66	9.124	0.010	a < c
Clinical Outcomes						
Phosphorus (mg/dl)	5.96 ± 1.89	5.32 ± 1.32	4.67 ± 1.48	15.957	0.000	a > b > c
Potassium (Eq/L)	5.12 ± 1.43	4.78 ± 0.84	4.51 ± 0.77	7.075	0.029	
Hemoglobin (g/dl)	10.74 ± 3.16	10.45 ± 0.99	10.81 ± 1.42	1.663	0.435	
Albumin (g/dl)	4.02 ± 0.57	3.94 ± 0.39	3.86 ± 0.52	4.578	0.101	

Mean scores with different subscripts differ significantly at *p* < 0.05 by the Bonferroni correction.

**Table 4:** Partial correlation between age groups, illness perception, self-care management, and clinical outcomes in hemodialysis patients (*n* = 189).

Variable	Univariate Correlation		Adjusted for Age Group	
	Age Groups	Illness Perception	Age Groups	Illness Perception
Self-Care Management	0.148(.043)	0.282(0.000)	-	0.268(0.000)
Phosphorus (mg/dl)	−0.284(.000)	−0.257(0.000)	-	−0.233(0.001)
Potassium (Eq/L)	−0.223(.002)	0.208(0.004)	-	0.244(0.001)
Hemoglobin (g/dl)	0.063(.393)	0.147(0.045)	-	0.140(0.056)
Albumin (g/dl)	−0.107(.146)	0.011(0.884)	-	0.024(0.741)

Correlation coefficient (*p*-value).
